# Divergent adaptive and innate immunological responses are observed in humans following blunt trauma

**DOI:** 10.1186/1471-2172-11-4

**Published:** 2010-01-25

**Authors:** Kevin R Kasten, Holly S Goetzman, Maria R Reid, Alison M Rasper, Samuel G Adediran, Chad T Robinson, Cindy M Cave, Joseph S Solomkin, Alex B Lentsch, Jay A Johannigman, Charles C Caldwell

**Affiliations:** 1 Division of Research, Department of Surgery, University of Cincinnati College of Medicine, Cincinnati, Ohio 45267-0558, USA

## Abstract

**Background:**

The immune response to trauma has traditionally been modeled to consist of the systemic inflammatory response syndrome (SIRS) followed by the compensatory anti-inflammatory response syndrome (CARS). We investigated these responses in a homogenous cohort of male, severe blunt trauma patients admitted to a University Hospital surgical intensive care unit (SICU). After obtaining consent, peripheral blood was drawn up to 96 hours following injury. The enumeration and functionality of both myeloid and lymphocyte cell populations were determined.

**Results:**

Neutrophil numbers were observed to be elevated in trauma patients as compared to healthy controls. Further, neutrophils isolated from trauma patients had increased raft formation and phospho-Akt. Consistent with this, the neutrophils had increased oxidative burst compared to healthy controls. In direct contrast, blood from trauma patients contained decreased naïve T cell numbers. Upon activation with a T cell specific mitogen, trauma patient T cells produced less IFN-gamma as compared to those from healthy controls. Consistent with these results, upon activation, trauma patient T cells were observed to have decreased T cell receptor mediated signaling.

**Conclusions:**

These results suggest that following trauma, there are concurrent and divergent immunological responses. These consist of a hyper-inflammatory response by the innate arm of the immune system concurrent with a hypo-inflammatory response by the adaptive arm.

## Background

Inflammation can be detected following trauma, even in the absence of infection, due to a global ischemia/reperfusion injury resulting in the systemic inflammatory response syndrome (SIRS). SIRS is diagnosed clinically when patients have more than one of the following clinical findings: significant changes in body temperature, tachycardia, tachypnea, or white blood cell count of >12,000 cells μL^-1 ^or <4,000 μL^-1^[1]. This hyper-inflammatory response can be further characterized by increased expression of inflammatory mediators such as pro-inflammatory cytokines, acute phase proteins, and complement that result in leukocyte activation and extravasation from the vascular compartment into surrounding tissues (reviewed in [2]). Additionally, numbers of neutrophils in the periphery increase, as does the priming of these cells [3,4]. Increased neutrophil numbers and activation can lead to bystander tissue damage with increased complications upon subsequent infection [5]. Recently, key structural elements of priming have been identified. Priming results in the formation of supramolecular complexes, which can include an array of receptors, structural proteins, and components of the nicotinamide adenine dinucleotide phosphate (NADPH) oxidase. These form on detergent-resistant membrane fragments called rafts, which are specialized membrane elements enriched in glycosphingolipids, cholesterol, and anti-apoptotic active Akt [6]. This proximity allows for close coupling of receptor mediated signaling, effector function, and crosstalk between surface receptors and downstream signaling systems [7].

The compensatory anti-inflammatory response syndrome (CARS) is proposed to follow SIRS in trauma patients. In the innate arm of the immune system, CARS can be characterized in patients exhibiting reductions in monocyte HLA-DR expression or ex vivo tumor necrosis factor alpha production [8]. In the adaptive arm of the immune system, CARS is characterized by a blunted response to infections due to decreased T cell numbers as well as mitogen unresponsiveness [9,10]. T cell apoptosis is responsible for declining T cell numbers following trauma [10,11]. Mitogen unresponsiveness after injury results in both reduced T cell cytokine production and decreased protein phosphorylation following T cell receptor-mediated stimulation [12,13]. Altogether, decreases in T cell numbers and responsiveness are thought to predispose trauma patients to nosocomial infections, sepsis, and multiple organ dysfunction [14,15].

These studies strongly suggest that trauma-induced changes to the immune system can predispose the patient to subsequent adverse clinical events, especially in the event of an infection. Currently, there are multiple therapies known to up or down regulate inflammation and the immune system [16]. Ideally, therapies should protect all cellular host defense compartments from hyper-inflammation, as well as from anergy. However, the response to trauma is dynamic and there exists a need to effectively monitor the immune status of patients in order to properly modulate inflammation.

In recent years there has been recognition that new approaches are needed for determination of the immune capacity of patients following trauma. Here, we enrolled a cohort of blunt trauma patients that was relatively homogenous in terms of sex, age, severity of injury, and mode of injury. We concurrently examined cells in both the innate and adaptive arms of the immune system in these patients in terms of absolute leukocyte numbers and function. Further, mechanisms underlying cell functionality were investigated. To our knowledge, this is the first report of a homogeneous cohort of trauma patients used to concurrently examine the function and mechanisms of both the innate and adaptive immune systems.

## Results

### Blunt trauma patient characteristics

There was no significant age difference between male blunt trauma patients enrolled in this study (36.3 ± 2.4 years) and healthy controls (37.8 ± 2.2 years). A mean Injury Severity Score (ISS) of 22.8 for all patients was calculated. 59% of patients requiring blood transfusion due to traumatic injury received an average of 1408 ml (± 552) packed red blood cells (PRBCs). 27% of patients were intubated at some point during their hospital stay (intubation strictly for operative intervention not included) and remained mechanically ventilated for an average of 14.2 (± 4.5) days. More than 85% of patients involved in this study underwent at least one surgical intervention. Those patients receiving at least one operation underwent an average of 2.6 (± 0.4) interventions during their admission. Total hospital length of stay for all patients in this study was 17.4 (± 2.2) days. Further characteristics of the patient population, including mean admission vital signs and lab values, are described in Table 1.

**Table 1:** Blunt Trauma Patient Characteristics

Age (Years)	36.3 ± 2.4
Injury Severity Score (ISS)	22.8 ± 2.1
Admission Temperature (°F)	97.9 ± 0.3
Admission Heart Rate	107 ± 5
Admission Systolic Blood Pressure	113 ± 5
Admission Diastolic Blood Pressure	72 ± 4
Admission Respiratory Rate	20 ± 1
Hemoglobin Concentration	9.0 ± 0.4
Platelet Count	145 ± 15
International Normalized Ratio (INR)	1.3 ± 0.1
Base Deficit	4.9 ± 1
Average Volume of PRBCs Transfused per Patient Requiring Transfusion (ml)	1408 ± 552
Average Number of Operations per Patient Requiring an Operation	2.6 ± 0.4
Average Number of Ventilator Days in Patients Requiring Intubation	14.2 ± 4.5
Hospital Length of Stay (Days)	17.4 ± 2.2

### Neutrophil numbers and function are increased following blunt trauma

**Figure 1:**
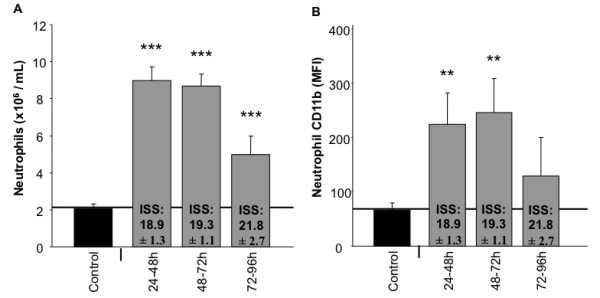
**Human circulating neutrophil numbers and activation are both increased following blunt trauma**. Results were obtained after collection of blood and flow cytometric analysis as described in the methods. (A) Absolute numbers of neutrophils are significantly increased following trauma. (B) Neutrophil activation is initially and remains increased following trauma. Data represents group size of 15 controls and 6, 7, and 9 blunt trauma patients at respective timeframes. *, p ≤ 0.05 versus controls. **, p ≤ 0.01. ***, p ≤ 0.001.

It has been observed that following trauma the proportion and absolute numbers of peripheral leukocytes can be altered. Here, in Figure 1a, we observed a 2-5 fold increase in circulating neutrophils out to five days post-trauma. Additionally, using β2-integrin (CD11b) as an activation marker, we observed significantly increased neutrophil activation as compared to controls (Figure 1b) out to four days post trauma. Thus, neutrophil numbers and activation are significantly higher following blunt trauma.

**Figure 2:**
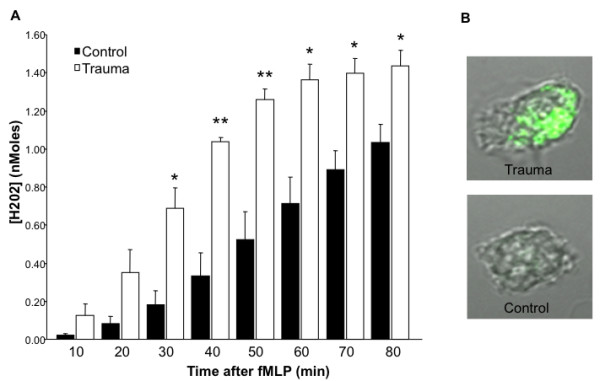
**Oxidative burst increased in blunt trauma following fMLP stimulation**. After specimen collection, neutrophils were stimulated and H_2_O_2 _production measured as described in the methods. (A) In comparison to controls, blunt trauma patients show increased oxidative burst activity. (B) Representative photographs of fMLP activated neutrophils were obtained demonstrating increased Dihydroxyrhodamine in a neutrophil from a blunt trauma patient at 48-72 h post injury. Data represents group size of 3 controls and 3 blunt trauma patients during the 48-72 h timeframe. *, p ≤ 0.05 versus controls. **, p ≤ 0.01.

Although we determined that neutrophils demonstrated an activated phenotype, we further investigated whether the neutrophils had increased functionality. Therefore, oxidative burst was evaluated in trauma versus control patients that were representative of those in Figure 1 in terms of ISS and age (Figure 2a). Here, neutrophils from the blunt trauma patients had a significant increase in fMLP-stimulated activity versus those neutrophils from controls. Additionally, we directly observed via confocal microscopy the increased oxidative burst in fMLP treated cells isolated from trauma patients as compared to healthy controls (Figure 2b). Thus, neutrophils isolated from trauma patients showed increased activation and functionality.

**Figure 3:**
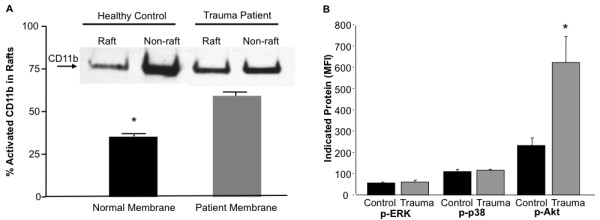
**Blunt trauma patients show increased percentage of activated CD11b within rafts, along with an elevated p-AKT**. (A) CD11b rafts of blunt trauma patients demonstrate a higher percentage of CD11b within rafts relative to controls with similar results demonstrated by western blot. (B) Phosphorylated (p)-AKT following blunt trauma is markedly elevated compared to controls, while p-ERK and p-p38 are similar between groups. Data represents group size of 4 controls and 4 blunt trauma patients during the 48-72 h timeframe. *, p ≤ 0.05 versus control.

Following trauma, there is an observed increase in pro-inflammatory cytokines, which may act to prime neutrophils. It has been reported that neutrophil priming involves the localization of activated CD11b within lipid rafts as well as increased phosphorylated Akt[7]. To investigate if this occurred within our cohort of patients, we isolated raft and non-raft fractions from purified neutrophils and probed for active CD11b (Figure 3a). Here, we found significantly more CD11b in neutrophil rafts from trauma patients as compared to controls. Additionally, we observed increased phosphorylated-Akt, but not -ERK or -p38, in neutrophils isolated form trauma patients (Figure 3b). These data indicate the neutrophils of trauma patients are primed, functionally more activated, and likely more resistant to apoptosis as compared to controls.

### T cell numbers and function are reduced following blunt trauma

**Figure 4:**
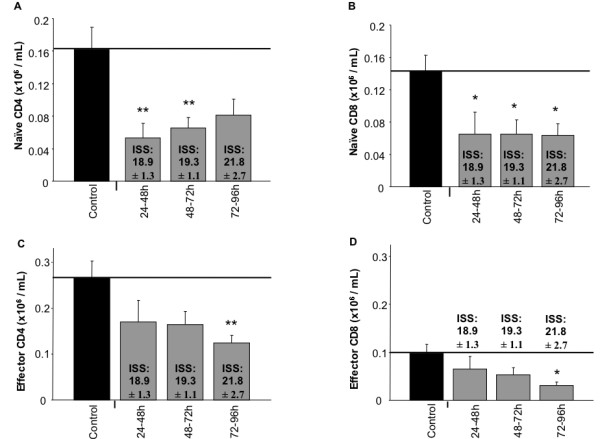
**Human circulating T cell numbers are decreased following blunt trauma**. Absolute numbers of (A) Naïve CD4, (B) Naïve CD8, (C) Effector CD4, and (D) Effector CD8 T cell populations were obtained by flow cytometric analysis as described in the methods section. Data represents group size of 15 controls and 6, 7, and 9 blunt trauma patients at respective timeframes. *, p ≤ 0.05 versus controls. **, p ≤ 0.01.

The studies above investigated the innate response of the immune system to blunt trauma. Here, we continued our investigations by concurrently examining the response of the adaptive immune system in the same cohort of male blunt trauma patients and healthy controls. Flow cytometric analysis revealed that absolute numbers of peripheral naïve CD4 and CD8 T-cells from trauma patients were significantly decreased as compared to controls (Figure 4a and 4b). Temporally, the naive subtypes were observed at approximately 50% of the control values. Additionally, significant differences were observed in non-naïve T cells, though percentage differences were less stark than those of the naïve T cell populations. Thus, in a homogeneous cohort of blunt trauma patients, peripheral T cell numbers are rapidly decreased and remain decreased for at least 4 days following injury.

**Figure 5:**
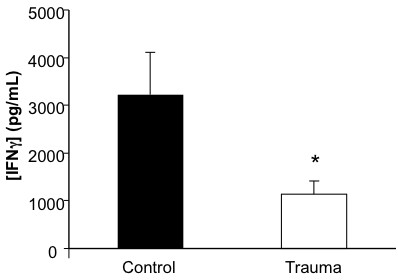
**IFN-γ production decreased in trauma patients**. Peripheral leukocytes were collected and then stimulated with cross-linked, soluble anti-CD3 and anti-CD28. After 48 hours, the cell culture supernatant was collected with IFN-γ concentration determined by ELISA. Data represents group size of 11 controls and 9 blunt trauma patients at 48-96 h post injury. *, p ≤ 0.05 versus controls.

IFN-γ is a potent activator of neutrophils and macrophages, and plays a key role in combating infections [18,19]. In our present study, we activated equal numbers of peripheral leukocytes isolated from blunt trauma patients 40-96 hours after injury together with age- and sex-matched controls using T cell specific mitogens for 48 hours and analyzed for IFN-γ accumulation (Figure 5). Here, we found trauma patients had significantly decreased IFN-γ production as compared to healthy controls.

**Figure 6:**
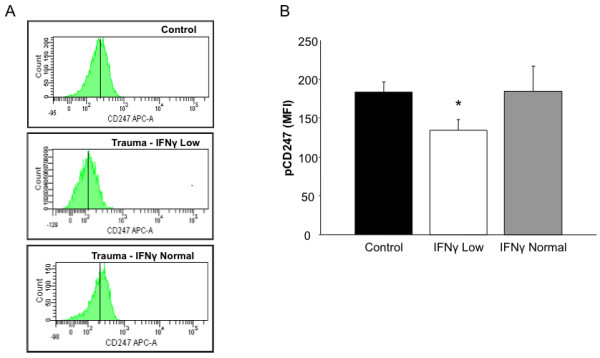
**The degree of CD247 phosphorylation is associated with bifurcated IFN-γ production in blunt trauma patients**. Representative flow cytometry (A) of CD247 phosphorylation in controls versus trauma patients with low and normal IFN-γ production. Mean fluorescence intensity (MFI) of CD247 phosphorylation (B) in control patients versus low-IFN-γ producers and normal IFN-γ producers. Data represents group size of 11 controls and 4-5 blunt trauma patients at 48-96 h post injury. *, p ≤ 0.05 versus controls.

In Figure 5, we show that T cells from trauma patients have reduced immune function. Previous reports have suggested that deficiencies in TCR-mediated signaling may result in decreased T cell-specific cytokine production [9]. The recent availability of phosphorylation-specific antibodies adapted for flow cytometry allowed us to determine on a single cell basis early activation events. The TCR-αβ or TCR-γδ and CD3, form the TCR-CD3 complex, which allows for antigen recognition and effective immune system functionality. The activation of this complex can be observed by the phosphorylation of immunoreceptor tyrosine-based activation motif (ITAM) tyrosines that are located on the TCR-ζ chain (CD247). Analysis of IFN-γ accumulation data demonstrated two distinct groups within our blunt trauma cohort that we stratified as those with low IFN-γ production and those not significantly different from healthy controls. We next wanted to determine whether these differences might be due to alterations in TCR mediated signaling. The phosphorylated CD247 (pCD247) expression was determined in these three subgroups (Figure 6). Here, we demonstrate that pCD247 expression was approximately 30% lower in the low IFN-γ producing group as compared to the other two groups. Thus, in patients whose T cell IFN-γ production is decreased, reduced TCR-mediated signaling is also demonstrated.

## Discussion

In our present study, we generated a cohort of trauma patients that was homogeneous in terms of sex, age, severity of injury, and mode of injury. Additionally, we concurrently investigated cells in the innate and adaptive arms of the immune system in this cohort of patients by determining the cell absolute numbers as well as function. Further, the mechanism underlying the function was determined. We believe that the combination of these three aspects make this a unique study.

In general, the immunological response to trauma has been modeled as a systemic inflammatory response followed sequentially by compensatory anti-inflammatory response. In our study, one aspect of SIRS, the increase in neutrophil priming and numbers, was observed up to 96 hours following trauma. One characteristic of CARS is reduced T cell numbers and impaired immune function. We also observed this phenomenon. However, this was seen almost immediately and up to 96 hours following trauma. Thus, aspects of SIRS and CARS were seen concurrently for a fairly prolonged period of time. Altogether, we believe that presence of both hyperactive neutrophils and anergic T cells needs to be considered when attempting to modulate the immune system following trauma so to beneficially modulate the subsequent response to adverse clinical events such as acute respiratory distress syndrome (ARDS), VAP and opportunistic infections.

This and previous studies [10] have demonstrated that trauma can lead to a decrease in the T cell numbers. While possible reasons for this decline include the effects from transfusion, dilution from resuscitation and movement from periphery, based on numerous studies, we feel apoptosis is the underlying cause of the decline [20,21]. The end result of massive naïve T cell apoptosis would include immunosuppression due to a loss of the TCR repertoire able to respond to subsequent infections, along with production of anti-inflammatory mediators by macrophages during phagocytosis of apoptotic bodies [22]. Further, the loss of T cell-mediated IFN-γ production serves to limit the activation of the innate immune system.

Initial events in TCR-mediated activation can have profound effects on T cell function. Within the TCR/CD3 complex are ITAMS which become phosphorylated during antigen-driven T cell activation. However, the degree of phosphorylation determines the degree of T cell activation. For example, a single ITAM phosphorylation activates SHP [23]. SHP expression is increased following trauma and its actions are known to be inhibitory [24]. Yet, two ITAM phosphorylations will result in full T cell activation [23]. Thus, we believe the differences observed in CD247 phosphorylation are, in part, responsible for the differences in IFN-γ production. Whether these differences are due to increased phosphatase activity or decreased gene expression is currently under investigation.

The T cell specific studies indicate that of the patients tested for IFN-γ production and CD247 phosphorylation, there were two fairly distinct groups. When compared to healthy controls, one group showed no significant differences in IFN-γ production and CD247 phosphorylation, while the other group showed significant decreases in each. Both groups had no significant differences in injury severity scores (25 versus 21, respectively) or age (32.4 versus 31.5 years, respectively). However, the hospital length of stay was trending towards an increase for those patients with poor T cell function (20.8 versus 12.3 days, p = 0.08). Whether or not these assays will be more predictive of adverse clinical events is an ongoing investigation.

Our data show that the function and numbers of neutrophils are increased following blunt trauma (Figure 1). Associated with this is increased phosphorylated Akt (Figure 3b). Active Akt is known to both decrease apoptosis as well as increase the oxidative burst [25,26]. The excessive number of these hyperactive cells can lead to excessive tissue damage, further worsening the condition of some trauma patients. Additionally, it has been shown that phosphorylated Akt can be prognostic for poor clinical outcomes [27]. We hypothesize that Akt has increased phosphorylation due to increased systemic IL-6 observed following trauma. Increased IL-6 can lead to increased tyrosine kinase activity by Jak-1 and -2 [28]. A potential tyrosine phosphorylation target of Jak-2 is PP2a [29]. When tyrosine-307 is phosphorylated, PP2a assembly as well as activity might selectively be inhibited [30]. This is important, as Akt is known to be de-phosphorylated by PP2a (reviewed in [31]). Although other mediators can activate Jak2, we believe IL-6 represents a likely candidate for future studies.

Although this study demonstrates new insights regarding the response of human T-cells to blunt trauma, it has limitations. First, we may not be measuring the whole of the immune compartment through our current methodology. For example, T cell numbers were not measured in the thymus, spleen or lymph nodes. Secondly, blunt trauma is wide-ranging and the effect of different mechanisms may play a role in the changes seen in this study. Even patients with non-significant differences in injury severity scores can have vastly different outcomes based on mechanism.

## Conclusion

In summary, our study demonstrates that there is a complex response of the innate and adaptive immune system quickly following trauma. We believe that this report distinguishes itself from other reports in that a very homogeneous cohort of patients was used. Additionally, both the innate and adaptive responses were numerically, functionally, and mechanistically examined concurrently. Altogether, these results demonstrate that there is a simultaneous and divergent immunological response to trauma. This consists of a hyper-inflammatory response by the innate arm of the immune system, while there is a hypo-inflammatory response by the adaptive arm. We believe that this concurrent divergence will need to be taken in consideration for therapeutic interventions.

## Methods

### Informed Consent/Patient Enrollment

In this study, 22 male blunt trauma patients and 15 healthy male subjects were enrolled over a period of 18 months (January 2007 through June 2008). Approval for human subject research was gained from the University of Cincinnati Institutional Review Board (protocol # 06-03-07-06). Written informed consent from the patient or patient representative and healthy control volunteers was received prior to obtaining blood samples. Patients were selected for inclusion based on the following criteria: (1) blunt trauma between the ages of 18 and 55, (2) admitted to the surgical intensive care unit, (3) no clinical evidence of immunosuppression and on no immunosuppressive medication, and (4) no clinical suspicion of sepsis.

### Peripheral Blood Isolation

Venous blood was drawn from patients and volunteer controls concurrently and placed into glass blood tubes with EDTA. Blood was drawn once at 09:00 am the day following enrollment. Serial blood draws from the same patient on subsequent days were not obtained. 6 patient samples were within the 24-48 h timeframe; 7 patient samples were within the 48-72 h timeframe; 9 patient samples were within the 72-96 h timeframe. Red blood cells were removed by placement of individual samples into lysis buffer (BD Pharmingen, San Diego, CA) for 10 minutes. After lysis, cells were prepared for flow cytometry as described below.

### Adherence Dependent Oxidative Response Assay

Ninety six-well polystyrene tissue culture dishes were coated with 1 μg/well fibronectin for two hours at 37°C and 5% CO_2 _and then washed. 100 μl of reaction mixture (10 mM scopoletin, 1 mg/ml horseradish peroxidase, 4 mM NaN_3 _in KRPG) and 20 μl neutrophils resuspended in KRPG at 7.5 × 10^5 ^cells/ml were allowed to incubate 10 minutes at 37°C in the wells prior to stimulation with either buffer or fMLP (100 nM). Fluorescence was measured immediately and at ten-minute intervals for 80 minutes. H_2_O_2 _production was calculated from the decrease in fluorescence due to the oxidation of scopoletin. The data are expressed as nanomoles of H_2_O_2 _produced per 1.5 × 10^4 ^PMNs.

### Adherence Dependent Oxidative Burst Imaging with Dihydroxyrhodamine

Peripheral leukocytes were loaded with dihydroxyrhodamine in HBSS in a 37°C water bath for 5 minutes. Cells were allowed to adhere to fibronectin coated μ-slide (ibidi-Integrated BioDiagnostics, Munchen, Germany) for 10 min at 37°C in 5% CO_2_. A 488 laser line with a band pass filter (505-550) was used to track the oxidation of dihydroxyrhodamine (Invitrogen, Carlsbad, CA) to rhodamine123. The image was captured with an inverted confocal microscope. Digital zoom equals 1.8×. After baseline images were captured 100 nm fMLP was added with recapture of images.

### Lipid Raft Fractionation and Western Blotting

PMN membrane protein (250 μg) was lysed for 30 min with 0.5 mls ice-cold 1% Brij 97 (Sigma-Aldrich, St. Louis, MO) plus 1 μg/ml leupeptin and pepstatin, 1 mM orthovanadate and 0.2 mM PMSF (Sigma-Aldrich, St. Louis, MO). The resulting lysate was mixed with 0.7 ml 80% sucrose and loaded into 4 ml Beckman SW 56 centrifuge tubes, overlaid with 2 ml of 35% sucrose, and 1 ml of 5% sucrose. Equilibrium centrifugation was performed at 200,000 × g, 4°C for 18 hours. Ten 0.4 ml fractions were collected from the top of the gradient. Lipid rafts were determined by dot blot analysis using HRP conjugated cholera toxin B (CTX-B). Equal volumes of lipid raft or non-raft fractions were resolved by 10% SDS-PAGE and transferred to nitrocellulose membranes. Membranes were probed overnight at 4°C with antibody to CD11b. Bands were visualized using chemiluminescence and autographic film.

### Leukocyte Identification and Enumeration by Flow Cytometric Analysis

Single-cell suspensions were prepared using standard procedures as previously described [17]. Cell counts were determined using a Beckman-Coulter AcT 10 cell counter. Cells were stained with the following: PB CD4 (Caltag, Carlsbad, CA), PB CD8 (Caltag, Carlsbad, CA), PerCP CD3 (BD Pharmingen, San Diego, CA), FITC CD45RA (Clone: JS-83; eBioscience, San Diego, CA), PE CD45RO (BD Pharmingen, San Diego, CA), PE CD11b (Clone: 1CRF44; eBioscience, San Diego, CA) and PB CD16 (Clone: 3G8, BD Pharmingen, San Diego, CA). CD4 and CD8 naïve subsets were defined by CD45RA-positive staining. CD4 and CD8 effector subsets were identified by CD45RO-positive staining. Flow cytometry data acquisition and analysis were performed on an LSR II using FACS Diva software (BD Pharmingen, San Diego, CA).

### Neutrophil phospho-Erk, -p38, and -Akt Expression Determined by PhosFlow Analysis

Neutrophils were stained with PE CD11b (Clone: 1CRF44; eBioscience, San Diego, CA) and FITC phospho-Erk (Thr202/Tyr204; Cell Signaling, Boston, MA), Alexa Fluor 647 phospho-p38 (T180/Y182; Cell Signaling, Boston, MA), or FITC phospho-Akt (Ser473; Cell Signaling, Boston, MA) fluorescently labeled antibodies. Flow cytometry data acquisition and analysis were performed on LSR II using FACS Diva software (BD Pharmingen, San Diego, CA).

### T-Cell phospho-CD247 Expression Determined by PhosFlow Analysis

T Cells were activated using soluble anti-CD3 (Clone: UCHT1; BD Pharmingen, San Diego, CA) and anti-CD28 (Clone: CD28.2; BD Pharmingen, San Diego, CA) cross-linked with secondary antibody for 5 minutes. Paraformaldehyde was used to stop the reaction. Cells were extensively washed and then solubilized with cold 90% methanol. The cells were labeled with PerCP CD3 (BD Pharmingen, San Diego, CA) and FITC phospho-CD247 (GenWay Bio, San Diego, CA) fluorescently labeled antibodies. Flow cytometry data acquisition and analysis were performed on LSR II using FACS Diva software (BD Pharmingen, San Diego, CA).

### T cell IFN-γ Production

Samples were stimulated with soluble anti-CD3 (Clone: UCHT1; BD Pharmingen, San Diego, CA) and anti-CD28 (Clone: CD28.2; BD Pharmingen, San Diego, CA) and cross-linked with secondary antibody for 48 hours. Culture supernatants were analyzed by ELISA for determination of IFN-γ (Peprotech, Rocky Hill, NJ).

### Statistical Analyses

Statistical comparisons were performed using either Student T Test or one-way ANOVA with Holm-Sidak post-hoc test utilizing SigmaStat 3.5 (Inspire Software International, Ashburn, VA). The mean and standard error of the mean were calculated in experiments with multiple data points. A value of P ≤ 0.05 was considered statistically significant.

## Authors' contributions

All authors read and approved the final manuscript. KK participated in acquisition and analysis of flow cytometric data, performed statistical analysis, and drafted the manuscript. HG participated in acquisition and analysis of flow cytometric data. MR participated in acquisition and analysis of flow cytometric data and IFNγ assays. AR participated in acquisition and analysis of flow cytometric data. SA participated in acquisition and analysis of flow cytometric data. CR participated in acquisition and analysis of confocal microsocopy photographs and lipid raft data. CC participated in acquisition and analysis of oxidative burst data. JS revised and provided critical input into the manuscript. AL revised and provided critical input into the manuscript. JJ revised and provided critical input into the manuscript. CC conceived of the study, participated in its design and coordination, and helped draft the manuscript. All authors read and approved the final paper.
